# Editorial: Rabies, a long-standing One Health example – progress, challenges, lessons and visions on the way to 0 by 30

**DOI:** 10.3389/fvets.2023.1220327

**Published:** 2023-07-18

**Authors:** Anna S. Fahrion, Conrad M. Freuling, Monique Léchenne, Thomas Müller, Sergio Recuenco, Marco A. N. Vigilato, Frank Busch, Kathrin Heitz-Tokpa, Stephanie Mauti, Mathew Muturi, Salome Dürr

**Affiliations:** ^1^Institute for International Animal Health/One Health, Friedrich-Loeffler-Institute, Federal Research Institute for Animal Health, Riems, Germany; ^2^Institute for Molecular Virology and Cell Biology, Friedrich-Loeffler-Institute, Federal Research Institute for Animal Health, Riems, Germany; ^3^Department of Epidemiology and Public Health, Swiss Tropical and Public Health Institute, Allschwil, Switzerland; ^4^Department of Public Health, Medical Faculty, University of Basel, Basel, Switzerland; ^5^Department of Preventive Medicine and Public Health, Faculty of Medicine, Universidad Nacional Mayor de San Marcos, Lima, Peru; ^6^Pan American Foot and Mouth Disease and Veterinary Public Health Center, Pan American Health Organization, Rio de Janeiro, Brazil; ^7^Centre Suisse de Recherches Scientifiques en Côte d'Ivoire, Abidjan, Côte d'Ivoire; ^8^Zoonotic Disease Unit, State Directorate of Veterinary Services, Nairobi, Kenya; ^9^Department of Veterinary Medicine, Dahlem Research School of Biomedical Sciences (DRS), Freie Universität Berlin, Berlin, Germany; ^10^Veterinary Public Health Institute, Vetsuisse Faculty, University of Bern, Bern, Switzerland

**Keywords:** rabies, veterinary public health, Zero by 30, One Health, disease elimination, neglected tropical disease (NTD), zoonosis

## Rabies control requires a One Health approach

Rabies is a zoonosis that is endemic in most African and Asian countries and has one of the highest case fatality rates of any disease. More than 95% of the estimated 59,000 annual cases of rabies in humans are attributed to dog bites (1, 2). Death and suffering from rabies are preventable, and the ambition to eliminate dog-mediated human rabies by 2030 has been formulated as “Zero by 30”, as manifested in a Global Strategic Plan by the United Against Rabies Coalition (3). However, even in regions that have made great strides in eliminating the disease, these successes remain fragile and hard to maintain if interventions are weakened or stopped because a country or region is no longer endemic (4).

One Health is an integrated, unifying approach that aims to sustainably balance and optimize the health of people, animals, and ecosystems and to generate incremental value by joining interdisciplinary forces. This view recognizes that the health of humans, domestic and wild animals, plants, and the wider environment (including ecosystems) are closely linked and interdependent (5). One Health approaches were proven to contribute to resilient communities and health systems long before the world was hit by the COVID-19 pandemic that brought One Health to the forefront of public attention. In the case of dog-mediated rabies, the need for close collaboration between the animal and human sectors has been evidenced and acknowledged for many years. Rabies experts have been emphasizing the importance of collaboration between sectors in surveillance, prevention, control, and elimination efforts even before the One Health concept became an increasingly common position of the “Tripartite” international organizations,^1^ in which rabies was given special relevance from the very beginning (6). The application of a One Health approach is also a central aspect of the global strategic plan towards elimination of dog-mediated human rabies not only as a means of successful rabies elimination but also because of the demonstrated economic savings.

This Research Topic called for papers presenting experiences, innovative perspectives, and visions for achieving the “Zero by 30” goal, with a special focus on the importance of One Health for rabies control, not only at the direct host-agent zoonotic interface but also considering environmental, sociological, and (geo)political aspects. A total of 147 authors responded to this call and contributed to 20 publications contained in this Research Topic, which include original research papers (15), brief research reports (1), perspectives (3), and opinion papers (1). The authors reported from the global (8) to the local level, with country and subnational examples from Benin (1), Brazil (2), Chad (2), Côte d'Ivoire (1), Indonesia (1), Mexico (1), Namibia (1), Thailand (2), and the Philippines (1).

## Political empowerment of the veterinary sector

The recent COVID-19 pandemic has given the One Health paradigm an unprecedented boost in public awareness, but all too often only in the direction of pandemic preparedness and response, to the detriment of endemic and neglected diseases. Nadal, Abela-Ridder, et al. provide insights into the disruptive effects of the COVID-19 pandemic on rabies control, highlighting that mass dog vaccination in 2020 was carried out as planned in only 5% of countries surveyed, access to post-exposure prophylaxis (PEP) decreased, and under-reporting worsened in many places. The authors conclude that veterinary services now need to take the lead in ensuring that they become an irreplaceable and integral part of public health services at local, regional, and global levels. Djegu et al. give an example of capacity strengthening in Benin's Central Veterinary Laboratory through the joint efforts of national authorities and inter- and non-governmental actors, leading to such empowerment of the veterinary sector.

## The importance of considering the cultural context

Social and cultural aspects play a paramount role when it comes to zoonotic disease prevention and control, notably for those that encompass close human-animal bonds, such as rabies. Nadal, Hampson, et al. highlight the importance of understanding the different cultural and religious contexts in which humans relate to animals. In particular, they refer to traditional approaches to treatment, such as faith healing, and religiously motivated attitudes toward rabies, i.e., accepting a disease as an expression of a divine will. N'Guessan et al. describe factors such as health-seeking behaviors and attitudes toward the concept of health and disease as determinants of rabies PEP dropout in the San-Pedro region of Côte d'Ivoire, and Mbaipago et al. emphasize, among others, religious motives such as the perceived impurity of the dog as barriers to treatment in the Republic of Chad. Premashthira et al. also take a look at the impact of socioeconomic factors on dog owners' knowledge, attitudes, and practices toward dog vaccination in Thailand.

## Improving dog vaccination, PEP, and rabies treatment

As has been demonstrated for decades, rabies can be fully prevented by vaccinating dogs (to eliminate it from the main reservoir population) and humans (to prevent the development of rabies after exposure). In a study from Thailand, Thanapongtarm et al. shed light on the characteristics of dog populations by describing the ownership correlation of free-roaming dogs. This knowledge can help to model dog populations with little effort and thus facilitate the planning of dog vaccination campaigns. The work of Lugelo et al. and Wera et al. is also dedicated to the facilitation and impact of mass vaccination of dogs by investigating the effectiveness of a passive cooling device on vaccine stability during a field study in Tanzania and by looking into factors influencing immunity in Indonesian dogs, respectively. Molini et al. focus on the complementary aspect of parenteral vaccination with oral rabies vaccines; the authors tested the immunogenicity of an oral vaccine strain in a field trial in Namibia.

In the context of human vaccinations, da Silva et al. report on an ongoing reliance on PEP in Brazil, despite the fact that dog rabies has not been eliminated and dog bite incidence remains high. As the use of PEP can be optimized by assessing the individual rabies risk of the bite victim, the authors advocate for closing knowledge gaps on PEP administration in collaboration with health professionals while improving communication between health and veterinary authorities. This points to the cross-sectoral concept of “integrated bite-case management” (IBCM), which aims to improve case detection and treatment, as showcased by Rysava et al. and Swedberg et al. While the former describes the potential of IBCM using an implementation case in Albay province, Philippines, the latter tackles the issue through a qualitative (interview) approach, confirming that IBCM needs to be embedded in the local context and cannot be implemented independently. Madjadinan et al. also used qualitative methods such as focus group discussions to illustrate barriers to PEP application following an IBCM approach and call for improvements in rabies health services and public awareness. Knobel et al. spin the One Health concept and look at a potential canine or human rabies therapy from a One Medicine perspective. They argue that little progress has been made in treating rabies-infected patients and plead for investigational combination therapy of naturally infected dogs as a model for a human clinical scenario. Using the experience gained, rabies could ideally be transformed into a treatable disease. This proposal has made the paper the most read in this Research Topic to date.

## The role of wildlife

Since 1980, thanks to the involvement of human health systems in mass canine vaccination campaigns, Latin America has made the greatest progress in the elimination of dog rabies, mainly through the governance mechanism of the National Rabies Program Directors (REDIPRA). In the majority of Latin American countries, there have been no human rabies deaths for years, mainly due to decades of mass dog vaccinations. Meanwhile, the focus of infection has shifted to another interface: the one between humans or domestic animals and wildlife reservoirs. Bats such as *Desmodus rotundus* remain vectors of rabies in countries like Brazil, as described by Megid et al., and Mexico, as highlighted by Ortega-Sánchez et al.. It is discussed that these species' epidemiological relevance for rabies transmission and elimination depends on various environmental factors, such as anthropogenic changes, and therefore requires continued surveillance. This underlines the importance of including the ecosystem as an integrated part of the One Health approach with regard to rabies control, particularly when rabies control in dogs is highly advanced.

## One Health solutions

This Research Topic comes at a time when the One Health concept is better understood and more “popular” than ever before, as countries strive to implement One Health strategies and link them with their mandated plans to eliminate rabies. This can break down silos and encourage governments, authorities, and other stakeholders to integrate the “systems approach” into their policies, resulting in shared expertise, joint action, and pooled resources. The United Against Rabies Forum (UARF) provides since 2020 a collaborative international network of partners from different sectors and disciplines to improve cross-sectoral coordination, reduce fragmentation, and support countries in their rabies elimination efforts. Tidman, Thumbi, et al. present the background, concept, and strategic direction of the UARF, while Tidman, Fahrion, et al. describe the composition of the Forum and the work carried out in the first 2 years of its existence to generate and compile approaches, tools, and materials to support countries in their efforts to achieve Zero by 30.

At local, regional, and global levels, we need more than ever a strengthened and integrated (veterinary, public, and environmental) health workforce to address challenges such as epidemics and climate change. Rabies control programs can contribute to building One Health capacity, opening opportunities to address other zoonotic threats, such as those with pandemic potential. Improving One Health coordination, collaboration, communication, and capacity building for rabies will contribute to this public good. To quote Ghai and Hemachudha, “It is time to target the political sector, to ensure that temporary disease burden reduction is not misconstrued as progress, to ensure that a legal framework is in place and that the strategies account for the restrictions imposed by the COVID-19 pandemic. It is crucial that countries maintain pressure and preserve the priority status of the disease at the country level, so that rabies can be eliminated once and for all” [SIC].

## Author contributions

AF, SD, CF, ML, TM, SR, and MV edited this Research Topic. FB, KH-T, SM, and MM supported the Research Topic as topic coordinators. AF and SD wrote this article, and all authors reviewed and complemented the manuscript and approved the submitted version.

## Footnotes

^1^The so-called Tripartite Collaboration, consisting of the Food and Agriculture Organization of the United Nations (FAO), the World Organization for Animal Health (WOAH, formerly OIE), and the World Health Organization (WHO), was joined in 2022 by the United Nations Environment Programme (UNEP) to form the “Quadripartite”.
